# Pathogenesis of vestibular schwannoma in ring chromosome 22

**DOI:** 10.1186/1471-2350-10-97

**Published:** 2009-09-22

**Authors:** Ellen Denayer, Hilde Brems, Paul de Cock, Gareth D Evans, Frank Van Calenbergh, Naomi Bowers, Raf Sciot, Maria Debiec-Rychter, Joris V Vermeesch, Jean-Pierre Fryns, Eric Legius

**Affiliations:** 1 Department of human genetics, University Hospital Gasthuisberg, Leuven, Belgium; 2 Department of Paediatrics, University Hospital Gasthuisberg, Leuven, Belgium; 3 Genetic Medicine, Manchester Academic Health Science Centre, St Mary's hospital, Manchester, UK; 4 Department of Neurosurgery, University Hospital Gasthuisberg, Leuven, Belgium; 5 Department of Pathology, University Hospital Gasthuisberg, Leuven, Belgium

## Abstract

**Background:**

Ring chromosome 22 is a rare human constitutional cytogenetic abnormality. Clinical features of neurofibromatosis type 1 and 2 as well as different tumour types have been reported in patients with ring chromosome 22. The pathogenesis of these tumours is not always clear yet.

**Methods:**

We report on a female patient with a ring chromosome 22 presenting with severe mental retardation, autistic behaviour, café-au-lait macules and facial dysmorphism. Peripheral blood lymphocytes were karyotyped and array CGH was performed on extracted DNA. At the age of 20 years she was diagnosed with a unilateral vestibular schwannoma. Tumour cells were analyzed by karyotyping, array CGH and *NF2 *mutation analysis.

**Results:**

Karyotype on peripheral blood lymphocytes revealed a ring chromosome 22 in all analyzed cells. A 1 Mb array CGH experiment on peripheral blood DNA showed a deletion of 5 terminal clones on the long arm of chromosome 22. Genetic analysis of vestibular schwannoma tissue revealed loss of the ring chromosome 22 and a somatic second hit in the *NF2 *gene on the remaining chromosome 22.

**Conclusion:**

We conclude that tumours can arise by the combination of loss of the ring chromosome and a pathogenic *NF2 *mutation on the remaining chromosome 22 in patients with ring chromosome 22. Our findings indicate that patients with a ring 22 should be monitored for NF2-related tumours starting in adolescence.

## Background

Ring chromosome 22 [r(22)] is a rare human constitutional abnormality. A distinct characteristic phenotype has not been delineated, but frequently reported features are delayed motor development, severe speech disability, hypotonia and microcephaly. In addition growth retardation, ataxia and seizures or abnormal EEG can be observed. Dysmorphic features are variable and mostly mild. Epicanthal folds, full eyebrows and large ears have been reported most frequently [1-4]. Aggressive behaviour as well as autistic disorder and hyperactivity are relatively common [1,3,4]. Internal organ involvement is rather rare,[3] except for central nervous system malformations [2]. The variable clinical presentation in carriers of a r(22) has been attributed to variable breakpoints and dynamic mosaicism. In common with other ring chromosomes, r(22) is assumed to arise from breakage and subsequent fusion of both chromosome arms with concomitant loss of sequences distal to the breakpoints [5]. Whereas deletion of ribosomal sequences on 22p is unlikely to be of clinical significance, loss of critical genes on 22q as well as unmasking of recessive alleles by the deletion may be expected to contribute to the phenotype. In a large review of 35 reported cases 22q loss varied from less than 69 kb up to 10.2 Mb in size with a weak correlation between the phenotypic variables and deletion size [3]. The phenotype can further be affected by the continuously evolving mosaicism (dynamic mosaicism) that is caused by the mitotic instability of the ring chromosome. Sister chromatid exchanges during mitosis can lead to formation of dicentric or interlocked rings and subsequent aneuploidy or rearrangements within the chromosome [6].

Clinical features compatible with neurofibromatosis type 1 (NF1) and 2 (NF2) have been associated with r(22) [7-12]. Reported features included hearing loss, mental retardation, seizures, meningiomas, peripheral neurofibromas, peripheral schwannomas, spinal tumours and vestibular schwannomas. In most reported cases the tumoural phenotype is most suggestive of NF2. We report on a patient with a r(22) and signs of neurofibromatosis who presented with a unilateral vestibular schwannoma at the age of 20. In DNA extracted from peripheral blood lymphocytes the 22q deletion size was examined by array CGH analysis. Analysis of tumour tissue showed loss of the ring 22 and in addition a pathogenic *NF2 *mutation.

## Methods

### Cytogenetic studies

Cytogenetic analysis of G-banded metaphase chromosomes was performed according to standard cytogenetic procedures on peripheral blood lymphocytes, skin-biopsy derived fibroblasts and on a primary culture of Schwann cells from the vestibular schwannoma. Culture conditions for Schwann cells were as described by Rosenbaum et al. [13] and Serra et al. [14].

Fluorescent In Situ Hybridisation (FISH) studies were performed on skin-biopsy derived fibroblasts using probes for *NF1 *(cFF13 and cFB5D) [15].

Genomic DNA was extracted from lymphocytes and vestibular schwannoma using standard techniques. Array CGH at 1 Mb resolution was carried out as described previously [16]. In the first array CGH experiment genomic DNA of the proband was used versus female reference DNA, in the second experiment DNA from the vestibular schwannoma of the proband was used versus genomic blood DNA of the proband.

### Mutation analysis

*NF1 *mutation analysis was conducted as described previously [15]. Briefly cDNA was generated from mRNA and used to amplify the *NF1 *coding region from position 48 to 8464 in eight overlapping PCR reactions. Each PCR fragment was sequenced using a solid-phase sequencing protocol.

To identify mutations in the *NF2 *gene Meta-PCR amplification followed by direct sequence analysis of the whole coding sequence including the immediate splice donor and splice acceptor sites was performed. In addition the P044 NF2 multiplex ligation-dependent probe amplification (MLPA) kit (MRC Holland) which measures copy number of all 17 exons of the *NF2 *gene and promoter was used to identify deletions and duplications of the gene. Loss of heterozygosity (LOH) analysis was carried out using microsattelite markers NF2CA3 (located within intron 1 of the *NF2 *gene) and D22S268 (located 5 kB telomeric to *NF2*).

### Consent

Written informed consent was obtained from the patients parents for publication of this case report and any accompanying images. A copy of the written consent is available for review by the Editor-in-Chief of this journal.

## Results

### Case report

**Figure 1:**
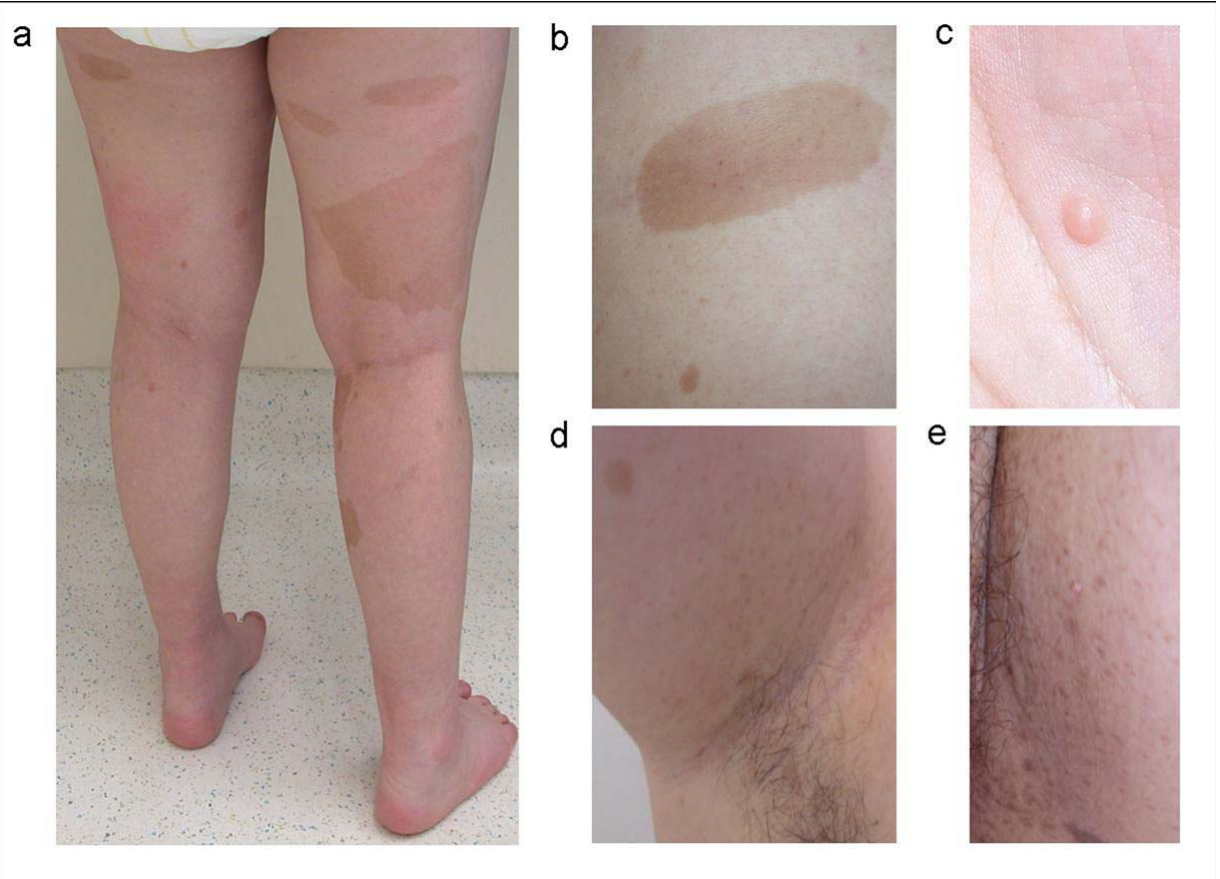
**Phenotypic features of patient with r(22)**. **(a) **Café-au-lait macules on the legs (b) Café-au-lait macule in the flank (c) Cutaneous nodule on the left hand (d) Axillary freckling (e) Inguinal freckling.

**Figure 2:**
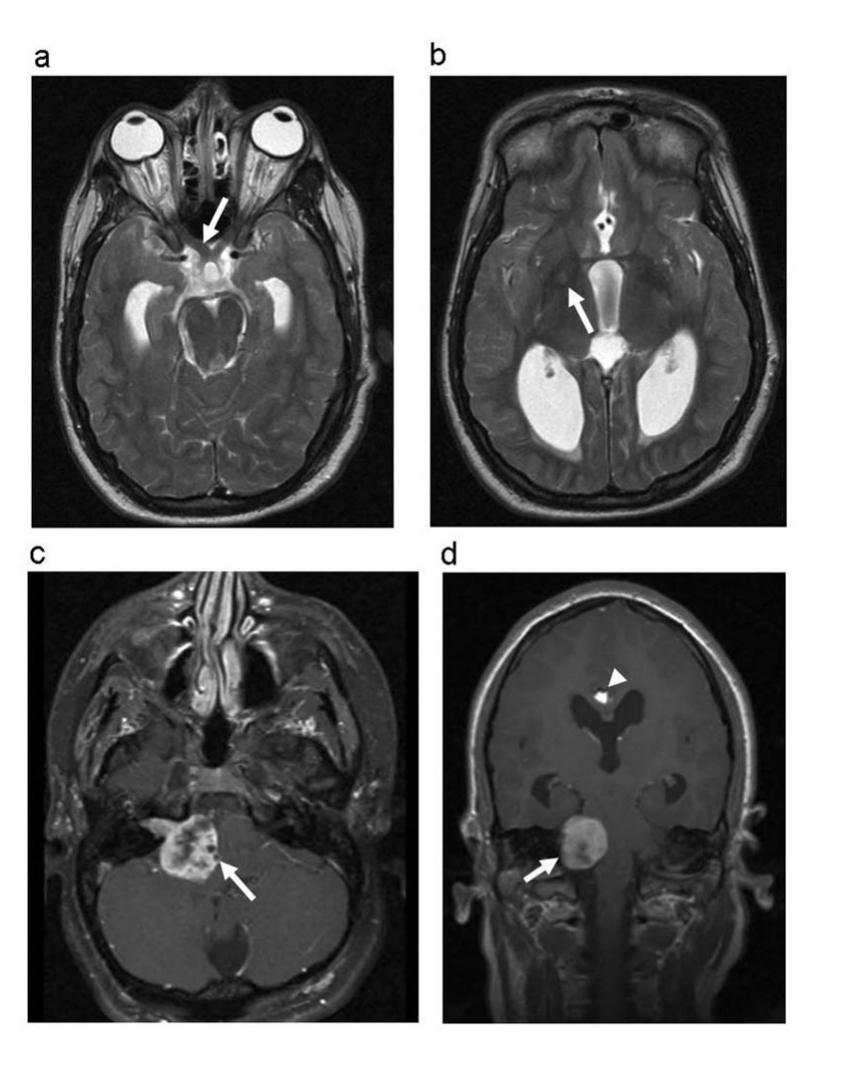
**MRI images of the brain at the age of 20 years**. (a) Horizontal T2 weighted section showing mild enlargement of the right optic nerve (white arrow) (b) Horizontal T2-weighted image showing a small hyper-intense spot in the right globus pallidus (arrow). Note also hydrocephalus. (c-d) Horizontal and coronal T1 weighted images showing the large partly cystic contrast enhancing schwannoma in the right cerebello-pontine angle. The tumoural mass causes deviation of the brain stem and the cerebellum to the left. (arrow) Note also supratentorial hydrocephalus and an interhemispheric lipoma (arrowhead).

**Figure 3:**
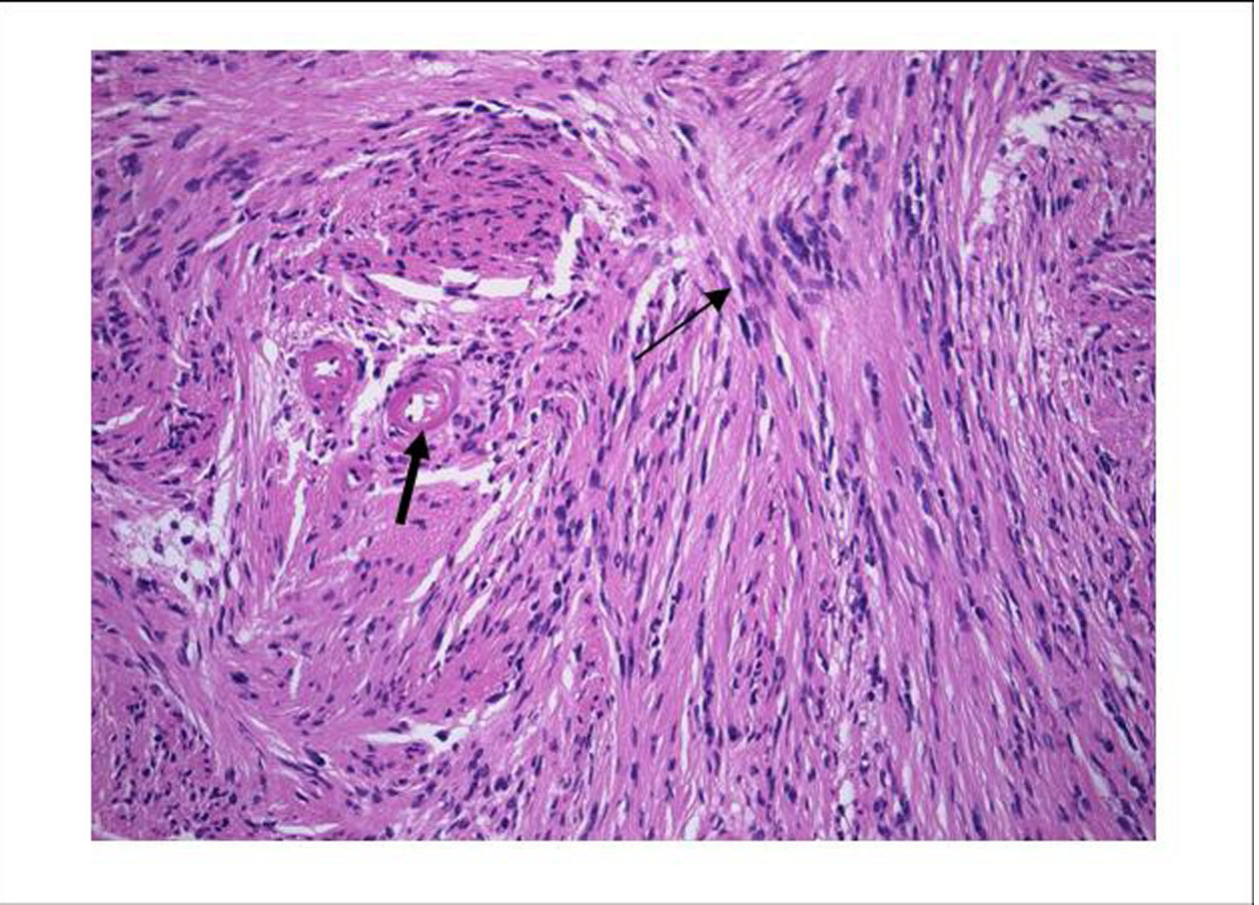
**Low power view of schwannoma**. Low power view of Schwannoma, illustrating the nuclear palisading (thin arrow) and the hyaline vessel walls (thick arrow). H&E stain, ×300.

The proband, a female, was born by caesarian section at the gestational age of 33 weeks. Pregnancy was complicated by preeclampsia. Her birth weight was 1730 g, length 42 cm and head circumference 30 cm. She could sit at the age of 1.5 years and walk at 2 years. She presented a lack of social contact. Until the age of 1.5 years she never smiled at people and refused eye contact. She was referred at the age of 2 years because of delayed psychomotor development, hypotonia and autistic features. Clinical examination showed multiple café-au-lait macules spread over the whole body and the diagnosis of NF1 was suspected (Figure 1a-b). At that time length and head circumference were at the 3^rd ^centile. During the following years she developed freckling in the axillary and inguinal region, and some cutaneous and subcutaneous nodules on the occipital region, the left hand and in the flank (Figure 1c-e). She was severely mentally retarded with lack of speech and toilet training and had difficult, autistiform behaviour with aggressive outbursts and automutilation. Dysmorphic features included a small forehead and low-set ears, large and broad hands and feet with short terminal phalanges. During teenage years her length was between the 3^rd ^and 25^th ^centile and head circumference between the 25^th ^and 50^th ^centile. She had hyperlordosis and bilateral pedes plani. Brain MRI at the age of 2 years showed a lipoma of the corpus callosum. Additionally at the age of 11 years a small T2 hyper-intense spot in the right globus pallidus as well as a mild enlargement of the pre-chiasmatic right optic nerve were present (Figure 2a-b). Evaluation of vision was difficult because of the mental retardation. At the age of 20 she presented with unsteady gait, right-sided paresis of the abducens nerve and bilateral papiloedema. Brain MRI showed a large tumour in the right cerebello-pontine angle with compression of the brainstem and cerebellum and obstructive hydrocephalus, suggestive of vestibular schwannoma (Figure 2c-d). The tumour was surgically removed and pathological examination confirmed the diagnosis of a vestibular schwannoma (Figure 3).

### Cytogenetic studies

**Figure 4:**
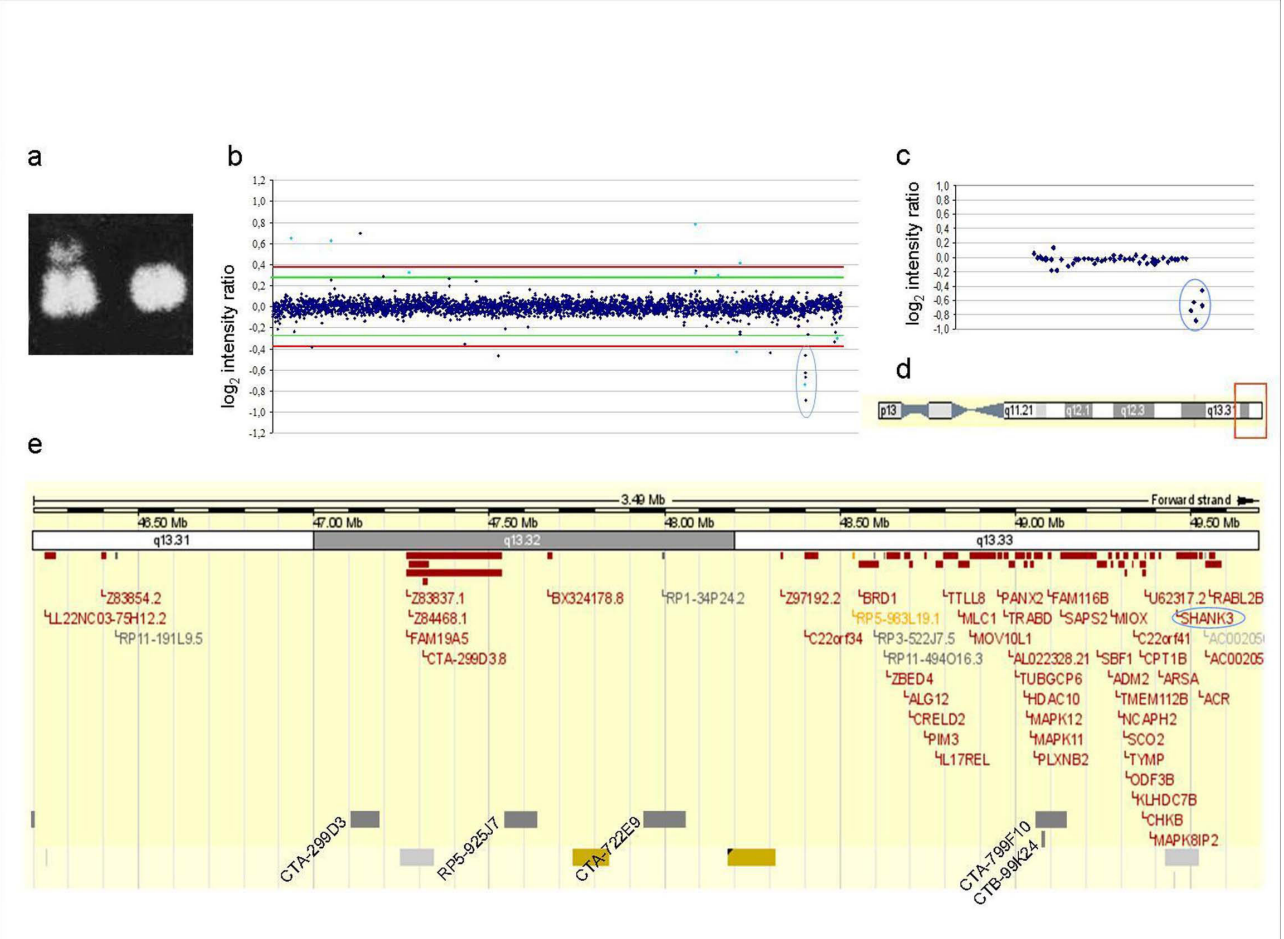
**Karyotype and array CGH on peripheral blood lymphocyte DNA**. (a) Detail of the constitutional karyotype of the patient showing the normal chromosome 22 and the ring chromosome 22. (b) Array CGH profile using a genome-wide micro-array with a 1 Mb resolution on genomic blood DNA of the proband and female reference DNA as the control sample, showing five consecutive clones with decreased copy number (encircled in blue). The Y-axis marks the hybridization ratio plotted on a log_2 _scale. Light blue dots indicate known polymorphic clones. The green and red lines indicate, respectively, the 4 × standard deviation [SD] threshold and the 0.58 - 2 × SD threshold [28]. (c) Detailed profile of chromosome 22 with deletion of five clones on the telomeric region of chromosome 22 (encircled in blue). (d) Overview of chromosome 22. The red empty square indicates the region shown in e. (e) Ensembl view for chromosome 22 (from 46 to 49 Mb) showing chromosome bands, Ensembl genes and 1 Mb clones. Deleted clones are indicated.

The constitutional karyotype of the patient was 46, XX, -22, + r(22) as revealed by cytogenetic analysis of peripheral blood lymphocytes and of skin-biopsy derived fibroblasts (Figure 4a). G-banded metaphases of primary Schwann cells from the vestibular schwannoma showed loss of the r(22) in all cells (n = 20) examined (karyotype: 45, XX, -22). FISH experiments on skin-biopsy derived fibroblasts with probes for *NF1 *(cFF13 and cFB5D) were normal.

### Array CGH

#### Peripheral blood lymphocyte DNA

Five clones (CTA-299D3, RP5-925J7, CTA-722E9, CTA-799F10, CTB-99K24) were deleted in the telomeric region of chromosome 22q: del 22(q13.32 → qter), comprising a region of approximately 2,5 Mb in size (47,248,304-49,453,810; Ensembl database, Release 52) (Figure 4b-e). This region also comprises the *SHANK3/PROSAP2 *gene, which has been proposed as a candidate gene for the abnormal brain development and autistic features in patients with r(22) or 22q deletion syndrome [17,18].

#### vestibular schwannoma

**Figure 5:**
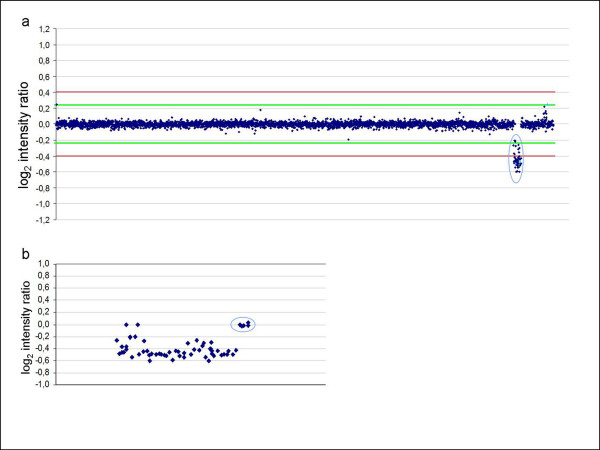
**Analysis of tumour tissue**. (a) Profile of array CGH experiment (1 Mb resolution) on DNA from vestibular schwannoma with DNA from peripheral lymphocytes of the proband as a control sample, showing deletion of clones on chromosome 22 (encircled in blue) except for five terminal clones. (b) This is more clear in the detailed view of chromosome 22.

Array CGH on DNA extracted from the vestibular schwannoma as the test sample and from peripheral blood of the proband as control showed loss of all clones on chromosome 22 except for the last 5 clones on 22q, confirming the loss of the ring chromosome (Figure 5).

### Mutation analysis

Sequencing of the *NF1 *gene did not show any pathogenic mutations. *NF2 *mutation analysis was performed on DNA from both peripheral blood and the vestibular schwannoma. There were no *NF2 *sequence abnormalities, intragenic deletions or copy number variations in blood. In the tumour DNA an *NF2 *point mutation was identified in a highly conserved nucleotide within the intron 14 splice donor site (c.1122+2T>A). MLPA and LOH analysis with two *NF2 *linked microsatellite markers (D22S268 and NF2CA3) showed loss of one copy of the *NF2 *gene in the vestibular schwannoma. This reflects the loss of the r(22) in the tumour tissue as seen by karyotyping and array CGH.

## Discussion

This is the first report describing the complete pathogenic sequence of tumour formation in a patient with r(22) with loss of the ring chromosome in the tumour tissue and a second hit in the *NF2 *gene on the remaining chromosome 22.

The karyotype and array CGH showed that the ring chromosome 22 was lost in the vestibular schwannoma. In addition *NF2 *mutation analysis on the tumour tissue showed a somatic splice-site mutation on the remaining chromosome 22. Predisposition for vestibular schwannomas in carriers of r(22) can be explained by Knudson's two-hit model. Due to the mitotic instability the ring chromosome is prone to loss during somatic mitosis (first hit). A mutation at the *NF2 *gene on the remaining chromosome 22 (second hit) in cells that have become monosomic for this chromosome can result in tumour development. This mechanism was also suspected by Tsilchorozidou in a patient with r(22) presenting with multiple meningiomas and a unilateral vestibular schwannoma. They found two truncating mutations in meningioma tissue as well as loss of one copy of the *NF2 *gene by LOH analysis [12]. However they did not prove loss of the ring chromosome in the tumour tissue. Interestingly increased tumour development has also been observed in carriers of other constitutional ring chromosomes, i.e. r(11) and r(13). Moreover the types of malignancy reported in these patients are concordant with the chromosomal assignment of tumour suppressor loci associated with Wilms' tumour (chromosome 11) and retinoblastoma (chromosome 13).

*INI1 *is a tumour suppressor gene located on chromosome 22 centromeric to *NF2*. It is involved in the development of malignant rhabdoid tumours and germline *INI1 *mutations are present in some cases of familial schwannomatosis, a condition characterized by the development of multiple spinal, peripheral and cranial-nerve schwannomas in the absence of vestibular schwannomas [19]. It is possible that still other tumour suppressor genes are located on chromosome 22 [20]. Moreover the *CHEK2 *gene, which encodes a checkpoint kinase important for the cell's response to DNA damage, has been recognized as a multi-organ cancer susceptibility gene and is located on chromosome 22 about 1 Mb centromeric to *NF2*. Thus a second hit in these alternative tumour suppressor genes in r(22) carriers that already have lost the ring in a number of cells can result in other tumour types than those typically seen in NF2. Indeed there have been reports of r(22) carriers with development of multiple meningiomas, cutaneous tumours and one testicular tumour but without the typical bilateral vestibular schwannomas of NF2 [8,11,12].

The patient reported here had been initially followed with a clinical diagnosis of NF1 because of multiple café-au-lait macules, freckling, a mildly enlarged right optic nerve and several subcutaneous nodules resembling neurofibromas. However these clinical features do not point to a true NF1 phenotype. Skin pigmentation abnormalities have been reported in patients with r(22) [21,22] and café-au-lait macules have been associated with multiple ring chromosomes (7, 11, 12, 15, 17) [23-27]. In view of the r(22) abnormality we believe that the enlargement of the right optic nerve could be due to a meningioma rather than to an optic glioma.

## Conclusion

We conclude that tumours can arise by the combination of loss of the ring chromosome and a pathogenic *NF2 *mutation on the remaining chromosome 22 in patients with r(22). Our findings indicate that patients with a ring 22 should be monitored for NF2-related tumours starting in adolescence.

## Competing interests

The authors declare that they have no competing interests.

## Authors' contributions

ED, HB and EL designed the study. ED carried out array CGH. HB was responsible for cell culture. PDC, FVC, JPF and EL participated in clinical management of the patient. RS analyzed the tumour anatomopathologically. MDR and JV participated in cytogenetic studies. DGE and NB participated in mutation analysis, MLPA and LOH analysis. All authors read and approved the manuscript.

## Pre-publication history

The pre-publication history for this paper can be accessed here:


